# Standardkonzentrationen für Dauerinfusionen – Ergebnisse einer bundesweiten Befragung auf deutschen Erwachsenenintensivstationen

**DOI:** 10.1007/s00063-022-00940-6

**Published:** 2022-07-15

**Authors:** Lutz Kreysing, Christian Waydhas, Karl Peter Ittner, Sebastian Schubert, Irene Krämer

**Affiliations:** 1 Apotheke der Universitätsmedizin Mainz, Johannes Gutenberg-Universität Mainz, Langenbeckstraße 1, 55131 Mainz, Deutschland; 2 Klinik und Poliklinik für Chirurgie, Berufsgenossenschaftliches Universitätsklinikum Bergmannsheil Bochum, Bochum, Deutschland; 3 Medizinische Fakultät, Universität Duisburg-Essen, Essen, Deutschland; 4 Lehr- und Forschungseinheit Pharmakologie, Universitätsklinik Regensburg, Regensburg, Deutschland

**Keywords:** Kontinuierliche Injektion, Injektionsrate gesteuerte Dosierung, Arzneimittel bei Intensivpatienten, Bundesweite Befragung, Arzneimitteltherapiesicherheit, Continuous infusion, Standardized concentration, Intensive care therapy, Nationwide survey, Medication safety

## Abstract

**Hintergrund:**

Intensivpatienten erhalten zahlreiche Arzneimittel (AM) als Dauerinfusion appliziert. In Deutschland fehlt bisher für die als Dauerinfusion applizierten AM eine einheitliche Liste mit Standardkonzentrationen.

**Ziel der Arbeit:**

Ziel war es, bundesweit repräsentative Informationen zu Standardkonzentrationen von als Dauerinfusion mittels Spritzenpumpe oder Infusionspumpe applizierten AM auf den Intensivstationen zu gewinnen.

**Material und Methoden:**

Zur Ermittlung der Akzeptanz und Präferenz für Dauerinfusionen von ausgewählten AM wurde ein Fragenkatalog in einem online- Umfragetool entwickelt und von der DIVI an die jeweils verantwortlichen Leiter*innen von 1816 Intensivstationen versendet. Die Umfrage umfasste Vorschläge zu 59 AM mit insgesamt 73 Konzentrationen. Ergänzend konnten die Teilnehmer in Freitextfeldern eigene Vorschläge zu AM und entsprechenden Konzentrationen angeben. Die Häufigkeit der Verwendung der Arzneimittel als Dauerinfusion und der präferierten Standardkonzentrationen wurde bezogen auf die Zahl der Antworten pro Arzneimittel berechnet.

**Ergebnisse:**

Die Umfrage wurde von 312 (17 %) Intensivstationen beantwortet. Die Akzeptanzrate für das Prinzip der geschwindigkeitsgesteuerten Dauerinfusion in Standardkonzentrationen ist deutschlandweit sehr hoch, Die Top 10 bzw. 25 der vorgeschlagenen AM werden von über 90 % bzw. 50 % der Teilnehmer regelmäßig als Dauerinfusion eingesetzt. Für viele dieser AM konnte eine oder mehrere präferierte Konzentrationen identifiziert werden.

**Diskussion:**

Die Top-37-Arzneimittel und die präferierten Konzentrationen sind als Grundlage für eine bundesweit geltende Standardliste mit Standardkonzentrationen für Dauerinfusionen (in der Regel 50 ml) geeignet. Die damit befassten Fachgesellschaften können basierend auf den Umfrageergebnissen eine bundeseinheitliche Standardliste konsentieren.

## Abstract (en)

**Background:**

Continuous infusion of numerous parenteral medications is common practice in intensive care units (ICU). In contrast to some countries, there is a lack of clearly defined standardized concentrations in Germany, especially for high-risk medications designated for infusion therapy.

**Objectives:**

The goal was to collect representative data of standardized concentrations commonly used for continuous infusion in German ICUs. Results should be used to draft nationwide recommendations for standardized continuous infusions.

**Materials and methods:**

To determine the nationwide acceptance and preference for medications designated for continuous infusion, a questionnaire was developed and sent to the directors of 1816 ICUs in Germany. The questionnaire comprised suggestions of 59 medicinal products with 73 concentrations. In addition, participants could make their own proposals on medications and concentrations preferably used. Evaluation was performed with SurveyMonkey® and Microsoft® Excel®.

**Results:**

A total of 312 (17%) ICUs answered the survey. Data analyses indicate a very high acceptance rate for rate-controlled continuous infusion with standardized concentrations. More than 90% (50%) of participating physicians routinely use the top 10 (top 25) medicines listed for continuous infusion. For most medicines, one concentration could be identified.

**Conclusions:**

Our survey results generate a suitable basis for a nationwide list with standardized concentrations of medicines intended for continuous infusion (usually 50 mL). Publication of such a list by the corresponding expert committees is likely to be met with broad acceptance and implementation into clinical practice can be expected.

Es ist allgemein anerkannt, dass durch Standardisierung die Prozesse der Arzneimitteltherapie sicherer, effektiver und effizienter gestaltet werden. Erstmals wurde bei deutschen Intensivmedizinern eine Onlineumfrage zu Standardkonzentrationen bei Dauerinfusionen durchgeführt, um die relevanten Arzneimittel und passende Standardkonzentrationen zu identifizieren. In diesem Beitrag werden die Ergebnisse der Umfrage und eine Vorschlagsliste für eine bundesweite Vereinheitlichung von Dauerinfusionen vorgestellt.

Die Reduzierung von Arzneimittelrisiken und die Verbesserung der Arzneimitteltherapiesicherheit (AMTS) gehören seit vielen Jahren in den meisten hochentwickelten Gesundheitssystemen zu den erklärten Zielen [4, 17]. Als wesentliche Qualitätssicherungsmaßnahme wird die Anwendung von Arzneimitteln in Standardkonzentrationen empfohlen [6, 9]. Patienten auf Intensivstationen erhalten eine Vielzahl von Arzneimitteln, häufig auch simultan, als Dauerinfusion appliziert. Dazu gehören Insulin, Heparin, Amiodaron, Kaliumchlorid, Sedativa und Katecholamine, die über eine geringe therapeutische Breite und ein hohes Risiko für unerwünschte Wirkungen verfügen. Die Verordnung, Vorbereitung und Applikation der Dauerinfusionen stellt einen komplexen, risikobehafteten Medikationsprozess dar [6]. Medikationsfehler können aus fehlerhaften Berechnungen der Dosis, fehlerhafter Zubereitung (u. a. falsche Menge, falsches Volumen, falsches Zubehör, Hygienefehler) und fehlerhaften Einstellungen der Infusionspumpen resultieren. Das Risiko von Medikationsfehlern wird durch die Applikation in einheitlichen Standardkonzentrationen und Steuerung der patientenindividuellen Dosis über das applizierte Volumen pro Zeit deutlich reduziert [3, 14, 19]. Dies ist besonders relevant beim Einsatz von Zeitarbeitskräften, von neuen Mitarbeitern oder bei Aushilfe durch Mitarbeiter von anderen Stationen [16]. Vergleichbar zum DIVI-Standard für die Etikettengestaltung von Arzneimitteln [5] könnte es auch von der DIVI empfohlene Standardkonzentrationen für Dauerinfusionen geben.

In den USA, Großbritannien und Spanien sind für Hochrisikoarzneimittel, die als (Dauer‑)Infusion appliziert werden, nationale Standardkonzentrationen definiert und deren Nutzung empfohlen [1, 2, 11, 14, 15]. Auch in Australien und Neuseeland wird eine nationale oder zumindest regionale Standardisierung der Parenteraliaapplikation bei Intensivpatienten angestrebt, um die Sicherheit, Effektivität und Wirtschaftlichkeit zu verbessern [10]. In Deutschland ist die Verordnung von Dauerinfusionen in Standardkonzentrationen an einzelnen Kliniken etabliert. Kürzlich wurde für die „Standard-Perfusor-Liste“ einer Universitätsmedizin eine Akzeptanzrate ca. 90 % berichtet [12]. Vor diesem Hintergrund ist auch in Deutschland eine bundesweit gültige Liste von Standardkonzentration für Arzneimittel zur Dauerinfusion anzustreben.

Ziel der vorliegenden Arbeit war es, bundesweit repräsentative Informationen zu Standardkonzentrationen von als Dauerinfusion mittels Spritzenpumpe oder Infusionspumpe applizierten Arzneimitteln auf den Intensivstationen zu gewinnen. Daraus könnten nationale Empfehlungen abgeleitet werden, die zu einer Vereinheitlichung der Applikation und Verbesserung der Arzneimitteltherapiesicherheit dieser Arzneimittel führen sollen.

## Methodik

Zur Ermittlung der bundesweiten Akzeptanz und Präferenz für Dauerinfusionen von ausgewählten Arzneimitteln wurde ein Fragenkatalog im Umfragetool SurveyMonkey® (SurveyMonkey Corp., San Mateo, CA, USA) entwickelt. Ausgangspunkt war eine publizierte Standardliste aus einem deutschen Zentrum [12]. In der Literatur wurde nach zusätzlichen möglicherweise relevanten Arzneimitteln und Dosierungen mit häufiger Anwendung und hohem Risiko gesucht [1, 7, 10, 11, 14, 15, 18]. Aus diesen Daten wurde im Rahmen von 2 Delphi-Runden der endgültige Fragenkatalog definiert. An den Delphi-Runden waren die Autorengruppe dieser Arbeit beteiligt, die sich aus Intensivmedizinern und Repräsentanten der Sektionen „Qualität und Ökonomie“ und „Angewandte Pharmakotherapie“ der DIVI und Krankenhausapothekern aus dem Bundesverband Deutscher Krankenhausapotheker (ADKA) zusammensetzte. Die Auswahl der im Fragenkatalog angegebenen Arzneimittel und Konzentrationen erfolgte unter der Maßgabe, in Deutschland gebräuchliche und häufig als Dauerinfusion eingesetzte Arzneimittel sowie typischerweise verwendete Konzentrationen zu definieren.

**Figure 1:**
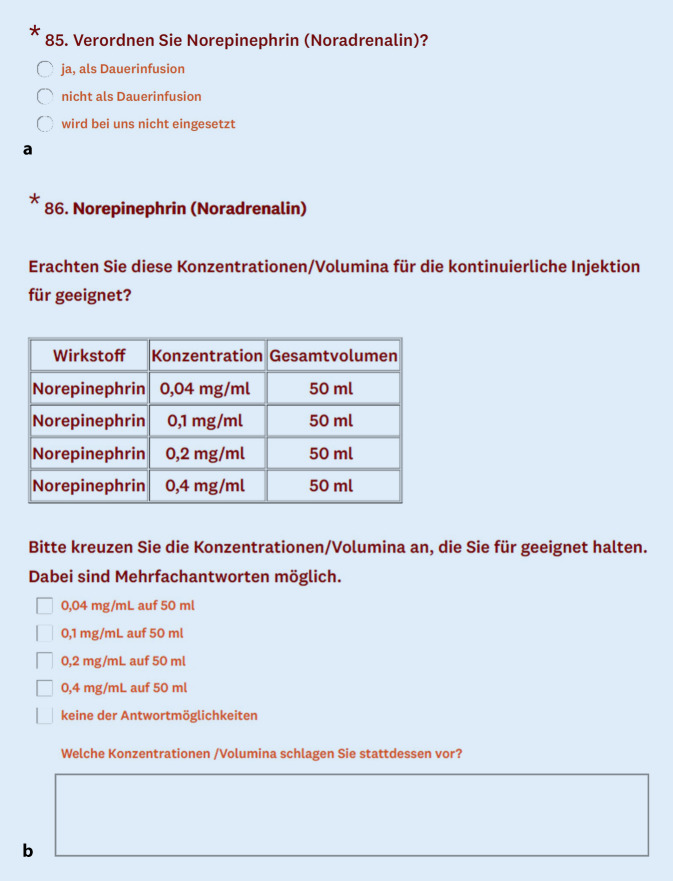

Der Fragenkatalog umfasste 59 Arzneimittel in alphabetischer Reihenfolge. Für jedes Arzneimittel wurde die Eingangsfrage gestellt, ob das betreffende Arzneimittel als Dauerinfusion eingesetzt wird (siehe Beispiel Norepinephrin Abb. 1a). Nur bei der Angabe „ja“ war die Frage nach der verwendeten Konzentration zu beantworten (siehe Beispiel Norepinephrin Abb. 1b). Neben den vorgegebenen Konzentrationen konnten im Freitext weitere Konzentrationen ergänzt werden, die auf der entsprechenden Intensivstation abweichend von den vorgeschlagenen Konzentrationen eingesetzt werden (s. Abb. 1b). Bei Verneinung der Eingangsfrage erfolgte die automatische Weiterleitung zum nächsten Arzneimittel.

Für 49 der 59 Arzneimittel wurde in der Umfrage eine Konzentration, für 8 weitere Arzneimittel 2 Konzentrationen und für 2 Arzneimittel 4 Konzentrationen angeboten. In der abschließenden Freitextfrage konnten die Teilnehmer zusätzliche als Dauerinfusion verabreichte Arzneimittel und Standardkonzentration(en) eintragen, die in ihrem Umfeld relevant sind oder häufig eingesetzt werden. Über 2 Testläufe wurde das Umfragetool in Bezug auf Bedienerfreundlichkeit optimiert.

Die Onlineumfrage wurde im April 2021 von der DIVI-Geschäftsstelle an den jeweils verantwortlichen ärztlichen Leiter jeder Intensivstation in der Adressdatenbank der DIVI versendet. Somit wurde von jeder Intensivstation eine Person kontaktiert und Dubletten vermieden. Im Verlauf erfolgten 2 Erinnerungen. Im Juni 2021 wurde die Umfrage geschlossen. Die statistische Auswertung der anonymen Umfrageergebnisse erfolgte mit SurveyMonkey® und mit Microsoft® Excel® (Version 2021) Microsoft Corporation, Redmond, WA, USA) für die Freitextantworten. Dabei wurde die Häufigkeit der Verwendung der Arzneimittel (als Dauerinfusion, Nichtdauerinfusion, keine Verwendung) und der präferierten Standardkonzentrationen bezogen auf die Zahl der Antworten pro Arzneimittel berechnet. Ein Votum einer Ethikkommission war nicht erforderlich, da es sich um keine biomedizinische Forschung mit Menschen bzw. mit deren Körpermaterialien/Daten am Patienten handelt (vgl. auch[8]). Das Einverständnis der befragten Ärzte wurde durch deren Teilnahme erteilt. Der Datenrücklauf war komplett anonymisiert, sodass keine Rückschlüsse auf die Teilnehmer möglich waren.

## Ergebnisse

An der Onlineumfrage nahmen insgesamt 312 (17 % der versendeten Einladungen) Erwachsenenintensivstationen teil. 166 e‑Fragebogen waren komplett ausgefüllt (9 % der versendeten Einladungen), 146 e‑Fragebögen (8 % der versendeten Einladungen) wurden partiell beantwortet.

**Figure 2:**
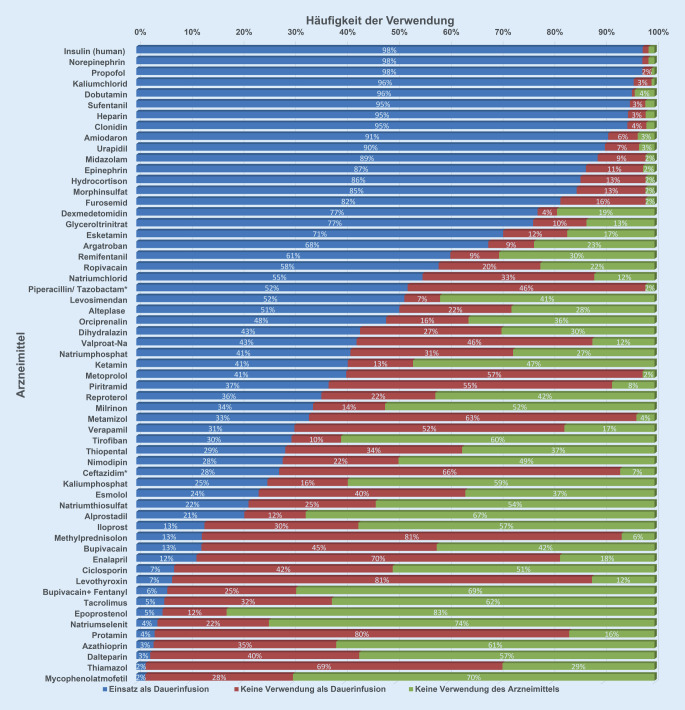

In Abb. 2 ist die prozentuale Häufigkeit der als Dauerinfusion verabreichten Arzneimittel in deutschen Erwachsenenintensivstationen in absteigender Reihenfolge graphisch dargestellt. Außerdem ist prozentual angegeben, wie häufig diese Arzneimittel als Nichtdauerinfusion oder überhaupt nicht auf den teilnehmenden Intensivstationen eingesetzt werden. Aus der Rangfolge ergeben sich die Top 10, Top 25 und Top 37 der verwendeten Arzneimittel.

Über 90 % der teilnehmenden Intensivstationen verordnen die Top-10- und über 50 % die Top-25-Arzneimittel als Dauerinfusion in Standardkonzentrationen. Darunter befindet sich auf Rang 23 das Antibiotikum Piperacillin/Tazobactam, das bereits von 50 % der teilnehmenden Intensivstationen als verlängerte Infusion angewendet wird. Die 12 Arzneimittel mit der Ranglistennummer 26–37 werden von mehr als 30 % der Umfrageteilnehmer als Dauerinfusion verordnet. Einige dieser Arzneimittel (z. B. Metoprolol, Piritramid, Metamizol, Valproat, Verapamil) werden häufiger als Kurzinfusionen angewendet. Die 22 Arzneimittel mit der Ranglistennummer 38–59 werden von weniger als 30 % der Umfrageteilnehmer als Dauerinfusion oder überhaupt verordnet. Dabei handelt es sich um Spezialarzneimittel wie Immunsuppressiva oder Periduralanalgetika. Im Freitext wurden als zusätzliche für die Dauerinfusion und Standardkonzentration geeignete Arzneimittel am häufigsten genannt: Vasopressinanaloga (Argipressin 0,8–1 IE/ml; Terlipressin 0,034–0,1 mg/ml), Pantoprazol 0,8–4 mg/ml, Magnesiumsulfat 100 mg/ml, Lormetazepam 0,08–2 mg/ml, Neostigmin 20–100 µg/ml, Lidocain 10–20 mg/ml, Meropenem 20–40 mg/ml und (Cis‑)Atracurium (1–2 mg/ml) 6 mg/ml.

In Tab. 1 sind die Top 37 als Dauerinfusion eingesetzten Arzneimittel zusammen mit den prozentualen Zustimmungsraten für die jeweilige Konzentration dargestellt. Für viele der 37 Arzneimittel gab es bei den präferierten Konzentrationen eine sehr große Übereinstimmung zwischen den Umfrageteilnehmern, wobei in der Regel jeweils eine Standardkonzentration die größte Akzeptanzrate erreichte (s. Tab. 1). Über 90 % Übereinstimmung ergab sich für die Dauerinfusion von Insulin 1 I.E./ml, Propofol 2 %, Kaliumchlorid 1 mmol/ml Dobutamin 5 mg/ml, Glyceroltrinitrat 1 mg/ml, Ropivacain 2 mg/ml, Piperacillin/Tazobactam 4,5 g in 50 ml und Alteplase 1 mg/ml. Von diesen stehen Propofol, Dobutamin, Gylceroltrinitrat und Ropivacain applikationsfertig als zugelassene Fertigarzneimittel zur Verfügung. Mehrere unterschiedliche Konzentrationen wurden insbesondere für Norepinephrin, Epinephrin, Amiodaron, Heparin und Levosimendan bevorzugt. Ergänzend sind in 2 weiteren Spalten der Tab. 1 zu Vergleichszwecken die Arzneimittel und Standardkonzentrationen aus der UK- und US-Standardliste aufgeführt [1, 15].

**Table 1:** 

Akzeptanzrate Standardkonzentrationen für 50 ml Dauerinjektionen bei erwachsenen Intensivpatienten			UK-Empfehlung 2020 [15]	USA-Empfehlung Update 2021 [1]
Arzneimittel zur Dauerinfusion in absteigender Häufigkeit der Anwendung	Standardkonzentration für 50 ml-Injektionsspritze	Akzeptanzrate für Konzentration (%)	Standardkonzentration für 50 ml	Standardkonzentration für 100 ml oder 250 ml
Insulin (human)	1 I.E./ml	94	1 I.E./ml	1 I.E./ml
Norepinephrin	0,04 mg/ml	14	–	–
	0,06 mg/ml	13	–	–
	0,1 mg/ml	72	0,08 mg/ml	0,016 mg/ml (250 ml)
	0,2 mg/ml	64	0,16 mg/ml	0,032 mg/ml (250 ml)
	0,4 mg/ml	17	0,32 mg/ml	0,128 mg/ml (250 ml)
	0,5 mg/ml	7	–	–
Propofol	20 mg/ml	91	–	–
	10 mg/ml	10	–	10 mg/ml
Kaliumchlorid	1 mmol/ml	98	–	–
Dobutamin	5 mg/ml	91	5 mg/ml	4 mg/ml
	10 mg/ml	13	–	–
Sufentanil	5 µg/ml	20	–	–
	10 µg/ml	58	–	–
	15 µg/ml	6	–	–
	20 µg/ml	10	–	–
Heparin	100 I.E./ml	5	–	–
	200 I.E./ml	46	–	–
	500 I.E./ml	43	1000 I.E./ml (20 ml)	100 I.E./ml
Clonidin	0,03 mg/ml	67	–	–
	0,015 mg/ml	20	0,015 mg/ml	–
Amiodaron	6 mg/ml	25	6 mg/ml	3,6 mg/ml
	18 mg/ml	28	18 mg/ml	–
	21 mg/ml	8	–	–
	24 mg/ml	36	–	–
Urapidil	5 mg/ml	84	–	–
	2 mg/ml	11	–	–
Midazolam	1 mg/ml	19	1 mg/ml	1 mg/ml
	2 mg/ml	77	2 mg/ml	–
Epinephrin	0,02 mg/ml	20	–	0,02 mg/ml (250 ml)
	0,06 mg/ml	12	–	0,04 mg/ml (250 ml)
	0,1 mg/ml	70	0,08 mg/ml	–
	0,12 mg/ml	5	–	–
	0,2 mg/ml	47	0,16 mg/ml	–
	0,4 mg/ml	12	0,32 mg/ml	–
Hydrokortison	2 mg/ml	68	–	–
	4 mg/ml	30	–	–
Morphinsulfat	2 mg/ml	56	2 mg/ml	5 mg/ml
	1 mg/ml	41	1 mg/ml	1 mg/ml
Furosemid	5 mg/ml	31	–	2 mg/ml
	10 mg/ml	68	–	10 mg/ml
Dexmedetomidin	4 µg/ml	17	4 µg/ml	4 µg/ml
	8 μg/ml	67	8 μg/ml	–
	20 μg/ml	26	–	–
Glyceroltrinitrat	1 mg/ml	96	–	0,2 mg/ml
Esketamin	25 mg/ml	84	–	–
Argatroban	0,2 mg/ml	29	–	–
	0,5 mg/ml	32	–	–
	1 mg/ml	47	–	1 mg/ml
Remifentanil	0,1 mg/ml	79	0,1 mg/ml	–
	0,02 mg/ml	8	–	–
	0,04 mg/ml	6	–	–
Ropivacain	2 mg/ml (200 ml)	97	–	–
Natriumchlorid	5,85 % (1-molar)	89	–	–
Piperacillin/Tazobactam	80 mg/ml bzw. 10 mg/ml	97	80 mg/ml bzw. 10 mg/ml (50 ml) 48 mg/ml bzw. 6 mg/ml (250 ml)	–
Levosimendan	0,25 mg/ml	50	–	–
	0,025 mg/ml (500 ml)	52	0,05 mg/ml (250 ml)	–
Alteplase	1 mg/ml	93	–	1 mg/ml
Orciprenalin	0,1 mg/ml	87	–	–
Dihydralazin	2 mg/ml	53	–	–
	1 mg/ml	38	–	–
Valproat-Na	24 mg/ml	65	–	–
	48 mg/ml	38	–	–
(Natrium)-Phosphat	(1 mmol/ml)-0,6 mmol/ml	86	0,4 mmol/ml	–
Ketamin	20 mg/ml	62	–	–
	50 mg/ml	23	–	–
Metoprolol	1 mg/ml	77	–	–
	0,5 mg/ml	19	–	–
Piritramid	1 mg/ml	90	–	–
Reproterol	9 μg/ml	70	–	–
	18 µg/ml	13	–	–
Milrinon	0,2 mg/ml	90	0,2 mg/ml	0,2 mg/ml
Metamizol	100 mg/ml	79	–	–
	50 mg/ml	7	–	–
Verapamil	1 mg/ml	92	–	–
Tirofiban	0,05 mg/ml (250 ml)	94	–	–

## Diskussion

Es ist allgemein anerkannt, dass durch Standardisierung die Prozesse der Arzneimitteltherapie sicherer, effektiver und effizienter gestaltet werden. Dauerinfusionen mit Standardkonzentrationen ermöglichen die elektronische Verordnung mit vorkonfigurierten Bausteinen, eine sichere Kommunikation bei Übergaben und Verlegungen sowie eine bessere Nachvollziehbarkeit der Vorbereitung und Etikettierung [13]. Applikationsfertige Infusionslösungen in Standardkonzentrationen vereinfachen zudem die Arzneimittellogistik, den Vorbereitungs- und Applikationsprozess.

Die Ergebnisse der bundesweiten Umfrage zu Dauerinfusionen zeigen eine hohe Akzeptanz für das Prinzip der Arzneimittelapplikation in Standardkonzentrationen und der geschwindigkeitsgesteuerten Dosisindividualisierung. Am höchsten ist die Akzeptanzrate und zudem die AMTS, wenn die Arzneimittel in der Standardkonzentration (z. B. Midazolam, Glyceroltrinitrat) in einem Standardvolumen von 50 ml als zugelassene oder von der Krankenhausapotheke eigenhergestellte Arzneimittel zur Verfügung stehen [12].

Gemäß der Umfrageergebnisse werden die Top-10-Arzneimittel von mehr als 90 %, die Top-25-von mehr als 50 % und die Top-37-Arzneimittel von mehr als 30 % der Umfrageteilnehmer als Dauerinfusion appliziert. Die niedrige Rate an Dauerapplikationen bei einigen Arzneimitteln (z. B. Ciclosporin und Tacrolimus) erklärt sich durch deren spezifische Indikation für Patienten, die nur von einem Teil der Intensivstationen behandelt werden. Auch bei den bevorzugten Standardkonzentrationen gibt es große Übereinstimmung, sodass die Top-37-Arzneimittel und die gewählten Konzentrationen für eine deutschlandweite Liste mit Standardkonzentrationen prädestiniert sind. Die in UK und den USA definierten Standards konzentrieren sich jeweils auf Arzneimittel, die überwiegend der Aufrechterhaltung der Vitalfunktionen bei den Intensivpatienten dienen. Nach unserer Erfahrung ist es sinnvoll, auch Arzneimittel mit speziellen Indikationen zu listen, um im Bedarfsfall die Information zur Verfügung zu haben. Die Unterschiede in den präferierten bzw. definierten Standardkonzentrationen resultieren aus unterschiedlichen Therapiegewohnheiten. Während in UK auch überwiegend 50 ml zur Dauerinjektion genutzt werden, werden in den USA in der Regel größere Volumen (100 ml, 250 ml) mit niedrigerer Konzentration infundiert (z. B. Katecholamine, Heparin). Die hohe Übereinstimmung bei den präferierten Standardkonzentrationen ergibt sich folgerichtig aus den zugelassenen Dosierungen mit Mengen pro kgKG und Zeiteinheit oder fixen Mengen pro Zeiteinheit und den zur Verfügung stehenden Fertigarzneimitteln. Andererseits müssen bei einigen Arzneimitteln (z. B. Amiodaron) verschiedene Konzentrationen für die zentralvenöse und periphervenöse Applikation zur Verfügung stehen.

Die Festlegung von Standardkonzentration folgt allgemein anerkannten Grundregeln [1]:

- falls das Arzneimittel als applikationsfertiges Fertigarzneimittel verfügbar ist, soll das Fertigarzneimittel in der zugelassenen Konzentration eingesetzt werden;
- wenn immer möglich, soll nur *eine* Konzentration als Standardkonzentration gewählt werden;
- zwecks Volumeneinsparung sollen soweit als möglich höher konzentrierte Lösungen eingesetzt werden;
- die gewählten Konzentrationen sollen zeit- und kostensparend aus den verfügbaren Fertigarzneimitteln vorzubereiten sein.

Diesen Grundregeln folgend wurde für die Top-37-Arzneimittel der Onlineumfrage ein Vorschlag für eine bundesweit geltende Standardliste mit Standardkonzentrationen für Dauerinfusionen (in der Regel 50 ml) erstellt. Die Vorschlagsliste mit infrage kommenden Arzneimitteln und Konzentrationen sowie Dosierungen sind Gegenstand von Tab. 2. Die Top-37-Liste wurde um 8 Arzneimittel (erkennbar an kursiver Schrift in Tab. 2) ergänzt, die in der Umfrage im Freitext zusätzlich angegeben worden waren oder von den Autoren als relevant eingestuft werden. Damit umfasst die Vorschlagsliste 45 Arzneimittel. Bei der überwiegenden Mehrzahl der Substanzen besteht bereits jetzt eine Präferenz für die Empfehlung einer einzigen Konzentration. Für eine Reihe von Arzneimitteln (z. B. Argatroban, Amiodaron, Heparin, Levosimendan, Morphin, Valproat) ist zu diskutieren, ob eine Empfehlung für 2 verschiedene Konzentrationen sinnvoll ist, um eine patientenindividuell adäquate Dosierung im Sinne der Praktikabilität (Infusionsvolumina, Häufigkeit der Spritzenwechsel u. a.) zu ermöglichen. Im Fall von Epinephrin und Norepinephrin müssen ggf. mehr als 2 empfohlene Konzentrationen in Betracht gezogen werden. Für Epinephrin werden als Konzentrationen 0,02 mg/ml, 0,1 mg/ml und 0,2 mg/ml, für Norepinephrin 0,1 mg/ml, 0,2 mg/ml und 0,4 mg/ml vorgeschlagen. In den USA wurden für Norepinephrin und Epinephrin bewusst unterschiedliche Standardkonzentrationen gewählt, um Verwechslungen der beiden Katecholamine zu vermeiden (z. B. Norepinephrin 0,016 mg/ml, Epinephrin 0,02 mg/ml; vgl. Tab. 1).

**Table 2:** 

Arzneimittel	Volumen 50 ml (wenn nicht anders angegeben) Standardkonzentration	Übliche Dosierung	Applikationsfertiges Fertigarzneimittel verfügbar
Alteplase	1 mg/ml	mg/h	Nein
Amiodaron	20 mg/ml	mg/h	Ja, 50 ml
Amiodaron	6 mg/ml	mg/h	Nein
Argatroban	1 mg/ml	µg/kg und Minute	Ja, 50 ml
Argatroban	0,5 mg/ml	µg/kg und Minute	Nein
*Atracurium*	6 mg/ml	mg/kg	Nein
*Cisatracurium*	2 mg/ml	mg/kg	Nein
Clonidin	30 µg/ml	mg/h; µg/h	Nein
Clonidin alternativ^b^	15 µg/ml	mg/h; µg/h	Nein
Dexmedetomidin	8 μg/ml	µg/kg und Stunde	Nein
Dexmedetomidin alternativ^b^	20 μg/ml	µg/kg und Stunde	Nein
Dihydralazin	2 mg/ml	mg/h	Nein
Dobutamin	5 mg/ml	µg/kg und Minute	Ja, 50 ml
*Enalapril*	0,05 mg/ml	mg/Tag	Nein
Epinephrin	0,02 mg/ml	µg/kg und Minute	Nein
Epinephrin	0,1 mg/ml	µg/kg und Minute	Nein
Epinephrin	0,2 mg/ml	µg/kg und Minute	Nein
Esketamin	25 mg/ml	mg/kg und Stunde	Ja, 50 ml
*Esmolol*	10 mg/ml (250 ml)	µg/kg und Minute	Ja, 250 ml
Furosemid	10 mg/ml	mg/h	Nein
Glyceroltrinitrat	1 mg/ml	mg/h	Ja 50 ml
Heparin	500 I.E./ml	IE/h	Nein
Heparin alternativ^b^	200 I.E./ml	IE/h	Nein
Hydrocortison	2 mg/ml	mg/h	Nein
Insulin (human)	1 I.E./ml	IE/h	Nein
Kaliumchlorid	1 mmol/ml	mmol/kg und Stunde; mmol/h	Ja, 50 ml
Ketamin	20 mg/ml	mg/kg und Stunde; mg/h	Nein
Levosimendan	0,25 mg/ml	µg/kg und Minute	Nein
Levosimendan	0,025 mg/ml (500 ml)	µg/kg und Minute	Nein
*Magnesiumsulfat*	100 mg/ml	g/h	Nein
*Meropenem*	20 mg/ml	g/Tag	Nein
Metamizol	100 mg/ml	mg/Tag	Nein
Metoprolol	1 mg/ml	mg/Tag	Nein
Midazolam	2 mg/ml	mg/kg und Stunde; mg/h	Ja, 50 ml
Milrinon	0,2 mg/ml	µg/kg und Minute	Nein
Morphinsulfat	2 mg/ml	mg/h	Nein
Morphinsulfat	1 mg/ml	mg/h	Nein
Natriumphosphat Kaliumphosphat	1 mmol/0,6 mmol/ml 1 mmol/0,6 mmol/ml	mmol/h mmol/h	Nein Nein
Natriumchlorid	5,85 % (1 mmol/ml)	mmol/h	Ja, 250 ml
Norepinephrin	0,1 mg/ml	µg/kg und Minute	Ja, 50 ml
Norepinephrin	0,2 mg/ml	µg/kg und Minute	Ja, 50 ml
Norepinephrin	0,4 mg/ml	µg/kg und Minute	Nein
Orciprenalin	0,1 mg/ml	µg/min	Nein
*Pantoprazol*	1,6 mg/ml	mg/h	Nein
Piperacillin/Tazobactam	80 mg/ml/10 mg/ml	g/Tag	Nein
Piritramid	1 mg/ml	mg/h	Nein
Propofol	20 mg/ml	mg/kg und Stunde; mg/h	Ja, 50 ml
Remifentanil	0,1 mg/ml	µg/kg und Minute	Nein
Reproterol	9 μg/ml	µg/kg und Minute	Nein
Ropivacain^a^	2 mg/ml (200 ml)	mg/h	Ja, 100 ml und 200 ml
Sufentanil	10 µg/ml	µg/kg und Stunde; µg/h	Ja, 50 ml
Tirofiban	0,05 mg/ml (250 ml)	µg/kg und Minute	Ja, 250 ml
Urapidil	5 mg/ml	mg/h	Nein
Valproat-Na	24 mg/ml	mg/kg und Stunde	Nein
Valproat-Na alternativ^b^	48 mg/ml	mg/kg und Stunde	Nein
*Vasopressinanaloga*			
*Argipressin*	0,8 IE/ml	IE/Tag	Nein
*Terlipressin*	0,034 mg/ml	µg/kg und Tag	Nein
Verapamil	1 mg/ml	mg/h bei > 50 kgKG mg/kg und Stunde bei < 50 kgKG	Nein

^a^Epidural und perineural, nicht intravasal

^b^Alternativ: Diskussionsgrundlage für Delphi-Verfahren

## Limitationen

Die Rücklaufquote von 17 % stellt die wesentliche Limitation der Studie dar, ist aber vergleichbar zu anderen Umfragen auf Intensivstationen [8]. Mögliche Gründe dafür könnten der Umfang des Fragenkatalogs oder die große Arbeitsbelastung auf den Intensivstationen in Folge der COVID-19-Pandemie sein. Andererseits handelt es sich unseres Wissens um die erste Studie in Deutschland, die Daten zur gelebten Praxis der Applikation von Arzneimitteln per Dauerinfusion auf Intensivstationen erhoben hat. Auch wenn die Rücklaufquote niedrig ist, gibt die absolute Zahl der Rückmeldungen ein hinreichendes Bild zu den Gepflogenheiten und Trends auf deutschen Intensivstationen. Eine Differenzierung der Ergebnisse nach Fachgebieten, Größe der Intensivstationen oder Art der Krankenhäuser (Grund‑, Maximal, Supramaximalversorger) ist aufgrund des Umfragedesigns nicht möglich. Ein Bias resultiert aus dem Anspruch, dass einheitliche Konzentrationen der Dauerinfusionen für alle Intensivstationen empfohlen werden sollten. Weiterhin ist es möglich, dass einzelne als Dauerinfusion verabreichte Arzneimittel oder gebräuchliche Konzentrationen in der Vorauswahl nicht berücksichtigt wurden. Die Umfrage ermöglichte jedoch die Angabe alternativ verwendeter Konzentrationen oder zusätzlicher Arzneimittel im Freitext. Genannt wurden 9 Arzneimittel, die bei zukünftigen Untersuchungen und Diskussionen berücksichtigt werden sollten.

## Ausblick

Für die Akzeptanz des Prinzips einer Standardliste mit Standardkonzentrationen ist es von Vorteil, wenn sie von den Fachgesellschaften initiiert, praxisorientiert und pragmatisch zusammengestellt sowie von mandatierten Experten entwickelt und empfohlen werden. Auf Basis der mit den Umfrageergebnissen erstellten Vorschlagsliste kann dieser Schritt von den Fachgesellschaften der Intensivmediziner und Krankenhausapotheker zeitnah durchgeführt werden. Somit könnte in Kürze in Analogie zu den „DIVI-Etiketten“ eine bundesweite Standardkonzentrationsliste für die Dauerinfusion bei erwachsenen Intensivpatienten etabliert werden. In einem nächsten Schritt ist es auch angebracht, die Datenbanken der Injektions‑/Infusionspumpen zu standardisieren und mit der elektronischen Verordnung („smart pump management“) zu verknüpfen.

Die Expertengruppe muss unter dem Aspekt der Akzeptanz den Umfang der Standardliste (weniger ist mehr, besser klein anfangen, mehr ist hilfreich) diskutieren. Bei der Publikation der endgültigen Liste sind die Gründe für die Aufnahme in die Liste und die Auswahl der Standardkonzentration darzulegen. Die Konzentrationen/Dosierungen können unter Umständen von den zugelassenen abweichen, aber durch neuere Studien, Leitlinien evident sein. Für Fälle eines formalen „off-label use“ ist eine Information oder Abstimmung mit den Zulassungsbehörden zu überlegen. In der Folge könnten auch die Zulassungsmodalitäten für applikationsfertige Fertigarzneimittel in Standardkonzentrationen und Volumen Gegenstand der Gespräche mit den Zulassungsbehörden und pharmazeutischen Unternehmern sein. Zu einem späteren Zeitpunkt wird es angebracht sein, die Akzeptanz in der Praxis zu überprüfen und ggf. Anpassungen vorzunehmen. Für die nationale Standardliste mit 16 Arzneimittelkonzentrationen in UK wurde 5 Jahre nach Einführung eine über 70 %ige Akzeptanzrate gefunden [14]. Da es sich um Empfehlungen handelt, können und müssen bei Bedarf für den individuellen Patienten abweichende Dosierungen gewählt werden.

## Fazit für die Praxis

- Die Akzeptanzrate für das Prinzip der geschwindigkeitsgesteuerten Dauerinfusion in Standardkonzentrationen ist deutschlandweit sehr hoch.
- Bei den bevorzugten Arzneimitteln und Standardkonzentrationen gibt es große Übereinstimmung.
- Für die überwiegende Zahl der Arzneimittel wird *eine* Standardkonzentration ausreichend sein; für Norepinephrin und Epinephrin werden je 3 Standardkonzentrationen vorgeschlagen.
- Die Top-37-Arzneimittel der Umfrage werden die Grundlage für eine deutschlandweite Liste mit Standardkonzentrationen bilden.
